# Effect of initiation treatment timing on efficacy of *Artemisia Annua* sublingual immunotherapy in patients with allergic rhinitis^☆^

**DOI:** 10.1016/j.waojou.2026.101381

**Published:** 2026-04-08

**Authors:** Jingyun Li, Dandan Fang, Tingting Ma, Yan Liu, Xu Zhang, Shixian Liu, Chengshuo Wang, Luo Zhang, Yuan Zhang

**Affiliations:** a Beijing Laboratory of Allergic Diseases and Beijing Key Laboratory of Nasal Diseases, Beijing Institute of Otolaryngology, Beijing 100005, China; b Department of Allergy, Beijing Tongren Hospital, Capital Medical University, Beijing 100730, China; c Department of Otolaryngology Head and Neck Surgery, Beijing Tongren Hospital, Capital Medical University, Beijing 100730, China; d Department of Allergy, Allergy Institute, Beijing Shijitan Hospital, Capital Medical University, Beijing 100038, China; e Department of Otorhinolaryngology, Head and Neck Surgery, Peking University People's Hospital, Beijing, China

**Keywords:** Allergic rhinitis, Sublingual immunotherapy, Efficacy, Time to treatment

## Abstract

**Background:**

Allergen specific immunotherapy (AIT) is a guideline-approved treatment that can modify the course of allergic rhinitis (AR). The treatment regimen for AIT in pollen-induced AR is more complex due to the impact of pollen exposure and there is no uniform recommendation on the time to initiate AIT for pollen-induced AR.

**Objective:**

The aim of this study was to investigate whether the initiation timing pre- or post-pollen season (PS) influence efficacy of *Artemisia Annua* sublingual immunotherapy (SLIT) in patients with allergic rhinitis.

**Methods:**

This was an open-label multicenter clinical trial. We recruited eligible patients in March 2022 and November 2022 from 3 treatment centers, and initiated SLIT before and after the PS of *Artemisia Annua* in 2022. Meanwhile, a control group was also set up to receive rescue medicine. Symptoms and medication usage were assessed throughout the PS, efficacy of SLIT was evaluated by comparing changes from baseline in: 1) the combined symptom-medication score (CSMS), 2) total nasal symptom score (TNSS), 3) ocular symptom score (TOSS), and 4) medication score (MS). A linear mixed-effects model (LMM) was employed to analyze the effects of treatment (SLIT vs. control), treatment initiation timing (pre-vs. post-season) on therapeutic outcomes.

**Results:**

SLIT significantly alleviated symptoms and reduced medication use compared to control, regardless of whether initiation before or after the PS. Furthermore, the LMM indicated that initiation timing (pre-vs. post-season) did not significantly affect treatment efficacy. There was no significant interaction effect between initiation timing and treatment group. It was also shown that higher pollen concentration was significantly associated with worse symptom severity and increased medication use.

**Conclusion:**

This multi-center study further confirms the efficacy of SLIT for *Artemisia Annua*-induced AR. We demonstrated that initiating SLIT before or after the PS did not affect its therapeutic efficacy by using LMM. This finding addresses a gap regarding the optimal timing for SLIT initiation and provides crucial evidence to inform clinical decision-making, offering greater flexibility in treatment scheduling.

**Clinical trial registrations:**

NCT05318157; Registered March 22, 2008.

## Introduction

Allergic rhinitis (AR) is a chronic inflammatory disease of the nasal mucosa mediated by specific immunoglobulin E (IgE),^1^ which has now become a global public health problem due to its high prevalence and significant burden on patients’ physical and mental health.^2^^,^^3^ Airborne pollen is one of the important allergens of AR,^4^ the global prevalence of pollen-induced AR (PIAR) is estimated to be 14.4%.^3^ A study of 6043 subjects in the grasslands of northern China revealed that 18.5% of these subjects had PIAR.^5^ Due to the global warming and more frequent weather emergencies, it has been shown that the symptoms and level of inflammation are more intensive and severe in PIAR.^6^^,^^7^ In addition, symptom improvement rates under standard drug therapy were significantly lower in patients with PIAR.^8^ Thus, management of PIAR requires special attention.

Allergen-specific immunotherapy (AIT) is the only treatment that could modify the immune response in AR.^9^ Previous studies have shown sublingual immunotherapy (SLIT) to be safe and effective for PIAR.^10^^,^^11^ Symptoms in patients with PIAR appear with exposure to allergenic pollen and its severity are associated with the level of pollen exposure.^12^^,^^13^ Due to the effects of pollen exposure, AIT regimens for PIAR patients are more complex and varied. Pre-seasonal, pre-coseasonal, and continuous regimens have been proposed in various trials.^14^, ^15^, ^16^ However, there are no publications comparing how the timing of SLIT initiation affects post-treatment outcomes. Our study explored the efficacy of *Artemisia Annua* SLIT and further compared the efficacy of PIAR allergic to *Artemisia Annua* with the initiation of SLIT at different time points.

## Methods

### Study population

This is a prospective, multicenter, clinical controlled study. Participants were recruited in March 2022 and November 2022 from the Department of Allergy at Beijing Tongren Hospital, Beijing Shijitan Hospital, and Peking University People's Hospital. The inclusion criteria were as follows:

(1) age between 18 and 60;
(2) a confirmed history of 2 or more years of PIAR in autumn with or without asthma and conjunctivitis;
(3) a positive *Artemisia Annua*-specific IgE level greater than 3.5 kU/L);
(4) the total nasal symptom score (TNSS) patients recalled during the last peak pollen season (PPS) reached or exceeded 6.

The TNSS is calculated by adding the scores from 4 nasal symptom evaluations: nasal itching, sneezing, rhinorrhea, and nasal obstruction. These symptoms are evaluated on a 4-point scale, ranging from 0, indicating no symptoms, to 3, signifying severe symptoms. Exclusion criteria included the presence of oral disease/oral allergy; a history of perennial AR, chronic rhinosinusitis, or immunosuppressive diseases; primary allergen is other types of autumn pollen allergen and living outside of Beijing during the PS. Those who have received pollen allergen-specific immunotherapy within the last 3 years, or who are currently receiving allergen-specific immunotherapy were excluded.

### Study design

**Fig. 1:**
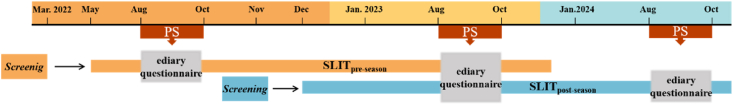
Study design. PS, the pollen season; SLIT, sublingual immunotherapy

Based on patients' personal preferences regarding the administration method, participants were divided into SLIT group and control group. The study design was illustrated in Fig. 1, patients recruited in March 2022 commenced treatment in May 2022, approximately 3 months prior to the onset of the PS. This cohort was designated as the pre-season group. Patients recruited in November 2022 initiated the administration of immunotherapy in December 2022, approximately 2 months following the PS, and this cohort was designated as a post-season group.

The SLIT schedule concluded 2 phases: the 5-week up-dosing phase, followed by a subsequent dose-maintenance phase. Patients retrieve the medication from the hospital and self-administer it sublingually on a daily basis. *Artemisia Annua* drops employed in the SLIT was produced from Zhejiang Wolwo Bio-Pharmaceutical, with ranging from 25 to 16 000 BU/mL (vial 1 ∼ vial 5). Vials 1–4 were used in the up-dosing phase, while vial 5 was employed in the maintenance phase. No intervention for the control groups. Patients in both the SLIT and control groups were permitted to use rescue medication, such as oral antihistamine, nasal corticosteroid spray, and short-term oral corticosteroid. Following 2 consecutive PSs, the pre-season group underwent efficacy assessment during the 2023 PS, while the post-season group was evaluated during the 2024 PS. During the PSs, patients recorded their daily symptoms (nasal and ocular) and medication use via daily electronic questionnaire completion.

### Pollen monitoring

*Artemisia Annua* is the most significant autumn pollen allergen in northern China, its pollen dispersing from July to October each year.^17^ Daily pollen concentration (grain/1000 mm^2^) was collected from Chinese pollen networks. The PS of *Artemisia Annua* is defined as the period from the first day of 3 consecutive days when daily pollen count was at least 100 grains per 1000 mm^2^ to the third day of 3 consecutive days when daily pollen count was less than 100 grains per 1000 mm^2^. Similarly, the PPS of *Artemisia Annua* is defined as the span between the first and last days when 300 grains per 1000 mm^2^ were counted.^18^

### Efficacy endpoints

The primary efficacy endpoint was the combined symptom medication score (CSMS) during the PPS. The CSMS (0–6) was calculated based on the same weighting of daily symptom score (dSS) (0–3) and daily medication score (MS) (0–3) for each day. The dSS assessed nasal (itchy nose, sneezing, runny nose, blocked nose) and ocular symptoms (itchy/red eyes, watery eyes), each of which was scored as 0–3 according to symptom severity; the MS was scored as 0–3 according to the grade of medication used. Secondary endpoints included TNSS, total ocular symptoms score (TOSS) and MS. The mean change from baseline of CSMS, TNSS, TOSS and MS were used to evaluate efficacy of the SLIT groups relative to control.

### Statistical analysis

We used the χ^2^ test for categorical variables and the Mann–Whitney *U* test or Student's t-test for continuous variables to assess the comparability of the baseline levels in each group and the efficacy of the SLIT relative to control. The above operations were performed using SPSS version 23.0. *P* < 0.05 was considered to be statistically significant.

We used a linear mixed-effect regression model (LMMs) with restricted maximum likelihood fitting to assess the effect on efficacy of the initiation timing for the CSMS, TNSS, TOSS and MS. Fixed effects included initiation timing (pre- or post-season), treatment (SLIT or control), daily pollen concentration and the treatment × initiation timing. Individual participants were treated as random effects. Continuous variables underwent z-score standardization, with pollen concentration undergoing dual standardization through logarithmic transformation and Z-score processing. For each SD elevation in the exposure variable, the fixed effect coefficient *β* estimated the incremental effect on the clinical endpoint. Construction of the models was conducted using the R package “lme4” “Matrix” “lmerTest”, with a significance level set at 0.05.

### Ethics committee

The study was approved by the Medical Ethics Committee of Beijing Tongren Hospital (TREC2022-KY029). All patients gave written consent to participate after being informed of the study's objectives and protocols.

## Results

### Study population

**Fig. 2:**
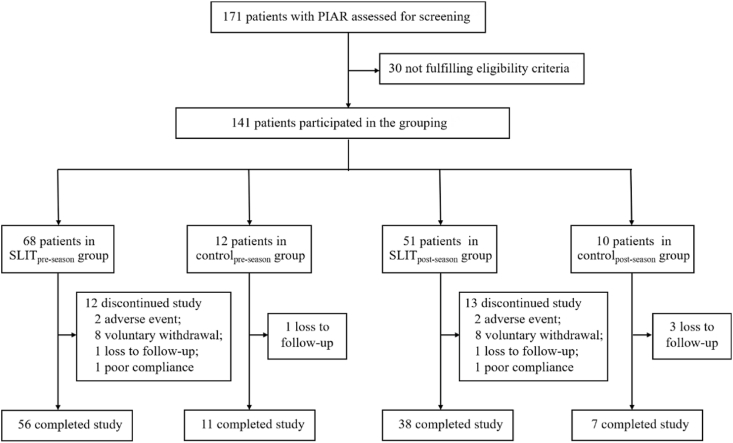
Diagram of patient enrollment. PIAR, pollen-induced allergic rhinitis; SLIT, sublingual immunotherapy

After screening 171 candidates with PIAR, 141 eligible patients were divided into 4 groups: SLIT_pre-season_ (n = 68), control_pre-season_ (n = 12), SLIT_post-season_ (n = 51), and control _post-season_ (n = 10). Finally, 82.4% (56/68) of participants in the SLIT_pre-season_ group, 83.3% (10/12) of participants in the control_pre-season_ group, 74.5% (38/51) of participants in the SLIT_post-season_ group and 70% (7/10) of participants in the control_post-season_ group completed the study. The flow diagram was shown in Fig. 2. There were no significant differences in baseline characteristics between the SLIT and control groups. The demographic and clinical characteristics of the patients in the groups are shown in Table 1.

**Table 1:** Demographic and clinical characteristics of study population.

Variables	SLIT (n = 94)	Control (n = 18)	*P* value	SLIT_pre-season_ (n = 56)	Control_pre-season_ (n = 11)	*P* value	SLIT_post-season_ (n = 38)	Control_post-season_ (n = 7)	*P* value
Age (years)	37.29 (9.63)	37.35 (7.77)	0.47	35.73 (8.42)	37.09 (9.61)	0.63	35.84 (8.44)	36.43 (4.54)	0.86
Gender, n (%)			1.00			0.95			1.00
Male	39 (41.49%)	7 (38.89%)		26 (46.43%)	5 (45.45%)		13 (34.21)	2 (28.57)	
Female	55 (58.51%)	11 (61.11%)		30 (53.57%)	6 (54.55%)		25 (65.80)	5 (71.43)	
BMI (kg/m^2^)	23.34 (3.42)	23.82 (3.05)	0.58	23.37 (3.36)	24.49 (3.11)	0.20	23.56 (3.54)	22.77 (2.86)	0.58
TNSS	9.29 (1.65)	9.11 (1.79)	0.66	9.34 (1.60)	9.00 (1.10)	0.36	9.26 (1.68)	9.29 (2.75)	0.98
Rhinorrhea	2.83 (0.41)	2.68 (0.58)	0.31	2.89 (0.37)	2.91 (0.30)	1.00	2.74 (0.45)	2.29 (0.76)	0.31
Nasal congestion	2.01 (0.69)	2.21 (0.85)	0.35	1.98 (0.73)	1.91 (0.83)	0.76	2.03 (0.64)	2.71 (0.76)	0.15
Nasal itching	2.16 (0.74)	2.00 (0.75)	0.39	2.18 (0.74)	2.00 (0.45)	0.31	2.16 (0.75)	2.0 (1.15)	0.05
Sneezing	2.29 (0.73)	2.21 (0.71)	0.65	2.29 (0.73)	2.18 (0.60)	0.54	2.34 (0.75)	2.29 (0.95)	0.62
TOSS	3.63 (1.33)	3.53 (1.33)	0.78	3.63 (1.47)	3.00 (1.70)	0.24	3.58 (1.13)	3.71 (1.38)	0.78
Ocular itching	2.38 (0.67)	2.19 (0.83)	0.51	2.37 (0.67)	1.60 (1.17)	0.07	2.39 (0.64)	2.43 (0.78)	0.89
Watery eyes	1.23 (0.88)	1.35 (0.80)	0.72	1.25 (1.06)	1.40 (0.70)	0.67	1.19 (0.82)	1.29 (1.11)	0.80
CSMS	3.91 (0.61)	3.82 (0.49)	0.55	4.01 (0.60)	3.89 (0.37)	0.53	3.78 (0.61)	3.74 (0.66)	0.86
MS	1.77 (0.45)	1.83 (0.51)	0.58	1.82 (0.43)	1.91 (0.30)	0.55	1.66 (0.48)	1.57 (0.53)	0.67
*Artemisia*-specific IgE (kU/L)	27.40 (25.33)	25.02 (23.20)	0.56	26.43 (24.48)	12.61 (21.91)	0.01	28.90 (26.90)	42.74 (9.57)	0.09

BMI, body mass index; CSMS, combined symptom-medication score; TNSS, total nasal symptom score; TOSS, total ocular symptom score; MS, medication score; SLIT, sublingual immunotherapy. Data were presented as n (%) or mean (standard deviation)

### Comparative efficacy of SLIT relative to control

**Fig. 3:**
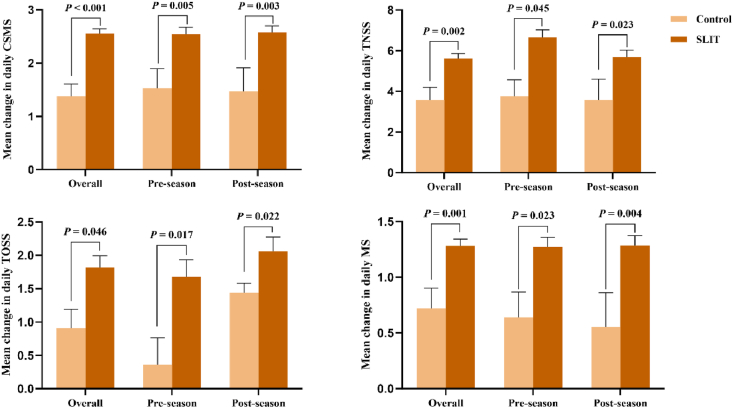
Comparative efficacy of SLIT relative to control. Compared to the control, *Artemisia Annua* SLIT significantly improved symptoms and reduced medication use across the entire study population and within subgroups (pre-season or post-season) stratified by initiation timing. CSMS, combined symptom-medication score; TNSS, total nasal symptom score; TOSS, total ocular symptom score; MS, medication score; SLIT, sublingual immunotherapy

**Fig. 4:**
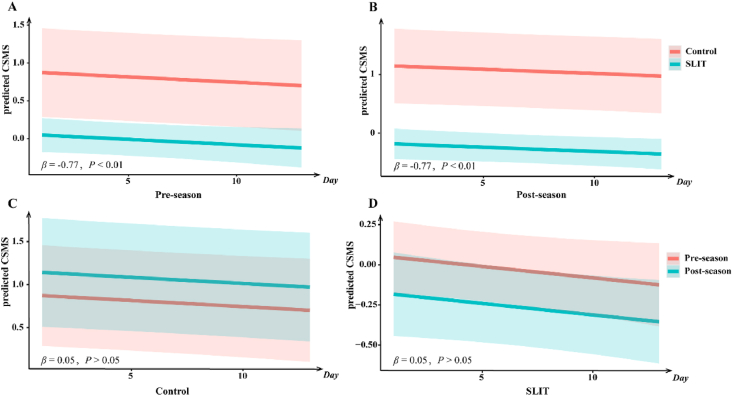
LMM-predicted CSMS over time for different groups (stratified by initiation timing and treatment). A/B: SLIT significantly improved the CSMS compared to the control in pre-season and post-season groups. C/D: There are no significant difference between pre-season and post-season group in SLIT or control groups. LMM, linear mixed model; CSMS, combined symptom-medication score; SLIT, sublingual immunotherapy

*Artemisia Annua* SLIT significantly improved patients' symptoms and reduced medication use compared to the control whether in the entire population or in the groups divided by initiation timing (all *P* < 0.05; Fig. 3). A total of 94 participants in the SLIT group and 18 participants in the control group completed the study. SLIT significantly decreased the change of mean daily CSMS [−2.55; (95% CI, −3.01, −2.05)] during PPS, compared with control [−1.38; (95% CI, −1.88, −0.89)]. Assessment of the effect on the secondary efficacy measures during the PPS demonstrated that SLIT also significantly decreased the changes of mean daily TNSS [−5.61 (95% CI, −6.12, −5.11)], TOSS [−1.82 (95% CI, −2.17, −1.48)] and MS [−1.28 (95% CI, −1.40, −1.15)] compared to control [TNSS = −3.57 (95% CI, −4.88, −2.26), TOSS = −0.91 (95% CI, −1.57, −0.25), MS = −0.72 (95% CI, −1.12, −0.33)] (see Fig. 4).

In the SLIT_pre-season_ group, the mean change from baseline was −2.54 (95% CI, −2.80, −2.28) for CSMS, −6.66 (95% CI, −7.91, −5.41) for TNSS, −1.68 (95% CI, −3.23, −0.12) for TOSS, and −1.27 (95% CI, −1.93, −0.98) for MS. Corresponding changes in the control_pre-season_ group were −1.52 (95% CI, −2.4, −0.65), −3.76 (95% CI, −5.69, −1.83), −0.36 (95% CI, −1.31, −0.59) and −0.64 (95% CI, −1.24, −0.14), respectively. Furthermore, the SLIT_post-season_ group [−2.57; (95% CI, −2.83, −2.32)] also demonstrated a significantly greater improvement in the mean change of CSMS scores from baseline compared to the control_post-season_ group [−1.47; (95% CI, −2.54, −0.39)]. Pronounced improvements were also observed in TNSS [SLIT_post-season_ = −5.69 (95% CI, −6.37, −5); control_post-season_ = −3.58 (95% CI, −6.07, −1.09)], TOSS [SLIT_post-season_ = −2.06 (95% CI, −2.50, −1.62); control_post-season_ = −1.44 (−1.78 to −1.09)], and MS [SLIT_post-season_ = −1.29 (95% CI, −1.46, −1.1); control_post-season_ = −0.55 (95% CI, −1.31, 0.2)]. These results indicate both pre-season and post-season SLIT groups demonstrated significantly greater improvements in all endpoints compared to their respective control groups.

### Efficacy comparison between pre-season and post-season SLIT groups

To investigate whether the initiation timing influences efficacy of SLIT, we used the data from the 2 groups which experimented 2 autumn PSs to construct LMMs. The results did not show any significant main effect of initiation timing (*P*_CSMS_ = 0.58, *P*_*TNSS*_ = 0.72, *P*_*TOSS*_ = 0.99, *P*_*MS*_ = 0.87) and treatment × initiation timing interaction (*P*_CSMS_ = 0.42, *P*_*TNSS*_ = 0.91, *P*_*TOSS*_ = 0.44, *P*_*MS*_ = 0.45). Compared to the control group, SLIT significantly improved the CSMS, TNSS, TOSS and MS during PS (group effect: CSMS *β* = −0.77, *P* < 0.01; TNSS *β* = −0.69, *P* < 0.01; TOSS *β* = −0.63, *P* < 0.01; MS *β* = −0.64, *P* = 0.01). Moreover, the findings indicate that elevated pollen concentrations were significantly correlated with increased symptom severity and greater medication use (CSMS *β* = 0.06, P < 0.01; TNSS *β* = 0.05, P = 0.02; TOSS *β* = 0.05, P = 0.02; MS *β* = 0.04, P = 0.04).

### Subgroup analysis

To investigate whether age influences the efficacy of SLIT, age was incorporated into LMM and to examine its potential interaction effect with treatment, the results as shown in Fig. S1–4. No significant effects were demonstrated for CSMS (*β* = −0.4, *P* = 0.08), TNSS (*β* = 0.31, *P* = 0.19), TOSS (*β* = −0.24, *P* = 0.32) or MS (*β* = −0.04, *P* = 0.14). The treatment × age interaction also illustrated no significantly effect on the average daily CSMS (*β* = 0.38, *P* = 0.11), TNSS (*β* = −0.45, *P* = 0.07), TOSS (*β* = 0.23, *P* = 0.38) or MS (*β* = 0.04, *P* = 0.14) within the SLIT group, as opposed to the control groups.

Additionally, a subgroup analysis stratified by sex was performed within the SLIT group. Among them, there were 48 males and 46 females. No significant differences in SLIT efficacy were observed between males and females (*P*_CSMS_ = 0.542, *P*_TNSS_ = 0.586, *P*_TOSS_ = 0.863, *P*_MS_ = 0.319), the results as shown in Fig. S5.

## Discussion

SLIT has been extensively studied in recent year, the efficacy and safety of SLIT for AR has been demonstrated in several studies.^19^^,^^20^ However, it is not clear when is the optimal time to initiate AIT for PIAR and whether the initiation timing influence efficacy of immunotherapy in *Artemisia Annua*-induced AR. To our knowledge, this is the first study to analyzed the effect of the timing of SLIT initiation on the efficacy of PIAR using LMMs. The study demonstrated that the timing of SLIT initiation had no significant impact on efficacy, which further confirms the robustness of SLIT efficacy and provides guidance on the timing of SLIT initiation in future.

Airborne pollens are the most common seasonal allergens in northern China.^21^ *Artemisia annua* is one of the most common autumn pollen allergens in PIAR.^17^ Lou et al conducted a randomized, double-blind, placebo-controlled, multicenter phase 3 clinical trial in which 702 patients with *Artemisia Annua*-induced AR were treated with SLIT or a placebo at a ratio of 2:1, and demonstrated the efficacy and safety of *Artemisia annua* extract drops.^18^ Recently, another study reported on the efficacy and safety of *Artemisia annua* SLIT in a Chinese pediatric population.^10^ Consistent with the findings above, this study demonstrated significantly greater improvements in CSMS, TNSS, TOSS, and MS from baseline in the SLIT group versus the control group. Furthermore, the effect of SLIT was also shown using LMMs, which is more accurate and reliable by taking into account the fixed effects of pollen concentration and the random effects of individual.

In this study, no effect of initiation timing and the treatment × initiation timing interaction on SLIT efficacy was found. Most studies initiate AIT before PS in previous PIAR-related studies.^22^^,^^23^ However, there is no consensus regarding the precise commencement date. A recent study evaluating the effect of different intervention times on the efficacy and safety of SLIT, and concluded that there was no difference in clinical efficacy and safety whether SLIT was initiated 8–9 weeks or 12–13 weeks before the season.^24^ Similar to these findings, our study supports flexible SLIT initiation timing. Crucially, we demonstrate that post-season initiation remains effective for symptom improvement and medication reduction. This provides valuable guidance for patients presenting after the PS who may hesitate to begin SLIT. In addition, there are some other regimens of administration for SLIT such as preseasonal, pre-coseasonal, coseasonal. it has been proven that a preseasonal period of minimum 8 weeks is effect for PIAR patients allergic to grass pollen, and prolonged preseasonal treatment (>8 weeks) improves efficacy of AIT during the PS.^25^ Some studies have initiated clinical trials within the PS and demonstrated the efficacy and safety of coseasonal initiation (CSI).^26^^,^^27^ And a meta-analysis proved that CSI doesn't increase risk of adverse event (AEs) of AIT.^28^ Stelmach et al. assessed the efficacy and safety of 2 SLIT regimens in grass pollen-allergic children: a pre-coseasonal regimen (initiated 8 weeks before the PS) and a continuous regimen (administered year-round). Both regimens demonstrated significant clinical benefits compared to placebo, with comparable reductions in the CSMS and most secondary endpoints.^16^ More clinical trials and health economic studies are needed in the future to identify the most applicable treatment regimen.

In the study, we observed a significantly positive impact of the pollen concentration on the average daily symptoms and medication use, corroborating previous findings.^29^^,^^30^ As evidenced in other studies, assessments the efficacy of AIT for patients with PIAR were significantly influenced by the level of pollen exposure. Durham et al. applied generalized additive modeling to quantify how AIT modulates the symptom-pollen concentration dynamic. Their analysis definitively established that the effect of AIT, especially during early-phase pollen exposure, is dependent on pollen concentration.^31^ In a five-year clinical trial on grass pollen immunotherapy revealed substantial interannual variations in pollen concentrations, with treatment efficacy demonstrating a pronounced dependency on exposure levels during PSs.^32^ Parallel findings emerged in a trial evaluating endolymphatic immunotherapy for grass/birch pollen allergy: while the active treatment group showed negligible clinical improvement after one-year treatment, subsequent PSs revealed progressively enhanced symptomatic relief and reduced medication usage. Investigators attributed the initial null outcome to differential pollen exposure between pre- and post-treatment monitoring periods.^33^ Pollen exposure should be considered into the evaluation of AIT efficacy, nevertheless, it remains systematically under-investigated in PIAR trials, and efficacy assessments rarely incorporate this key variable.^34^ The findings of the present study serve to underscore the significance of incorporating pollen exposure into the assessment of the efficacy of AIT. The exclusion of this critical factor may result in a diminution of the therapeutic efficacy of AIT.

There were some limitations in our study. First, patients were subjected to screening on the basis of retrospective mean scores from the last PS. Consequently, patients may recall only the period when symptoms were at their most severe, potentially leading to the inclusion of individuals with low scores in the study population. Such patients have been shown to demonstrate smaller values of improvement in scores after treatment, which may result in false-negative findings. Second, recruitment difficulties, particularly in maintaining a control group managed only with rescue medication, resulted in unequal group sizes between the SLIT and control groups, may have introduced potential bias. In addition, the duration of treatment differed between the 2 SLIT groups, with SLIT_post-season_ receiving treatment for a period nearly 6 months longer than SLIT_pre-season_. This is an unavoidable limitation due to the fact that efficacy must be observed during the PS, and the same number of PSs must be experienced. Nonetheless, previous research has demonstrated that the effectiveness of AIT can be predicted by early efficacy (after 6 months of treatment).^35^ From this perspective, the impact of the time discrepancy can be disregarded, as both groups received treatment for a duration exceeding one year.

## Availability of data and materials

All data generated or analyzed during this study are included in this published article.

## Authors’ contributions

Jingyun Li: Conceptualization, Writing- Original Draft. Dandan Fang: Data analysis, Writing- Original Draft. Tingting Ma: Supervision. Yan Liu: Methodology; Validation. Xu Zhang: Follow-up. Shixian Liu: Investigation. Chengshuo Wang: Supervision; Resources. Yuan Zhang: Project administration; Resources. Luo Zhang: Project administration; Resources.

## Ethics approval and consent to participate

Written informed content was received from each patient. This study received ethical approval from Beijing Tongren Hospital. Ethics approval: TREC2022-KY029. Clinical Trial Registration NCT05318157; Registered March 22, 2008.

## Consent for publication

The informed consent was obtained from study participants.

## Authors’ consent for publication

All the authors approved the final version for publication in WAO Journal.

## Declaration of generative AI and AI-assisted technologies in the writing process

Nothing to disclose.

## Funding

This work was supported by grants from Beijing Municipal Science & Technology Commission (Z211100002921060), national key R&D program of China (2022YFC2504100), the program for the Changjiang scholars and innovative research team (IRT13082), Natural Science Foundation of China (82471132 and 82071022) and Beijing Hospitals Authority Clinical medicine Development of special funding (ZLRK202303).

## Declaration of competing interests

No conflicts of interests, financial or otherwise, are declared by the authors.
