# Supplementary material for: Effect of initiation treatment timing on efficacy of Artemisia Annua sublingual immunotherapy in patients with allergic rhinitis
Source: World Allergy Organ J. 2026 Apr 8;19(4):101381. doi: 10.1016/j.waojou.2026.101381 (PMC13091189; doi:10.1016/j.waojou.2026.101381)
Supplement: Multimedia component 1 [file mmc1.pdf]

**Figure S1:** LMM-predicted CSMS over age for SLIT and control groups. LMM, linear mixed model; CSMS, combined symptom-medication score; SLIT, sublingual immunotherapy.

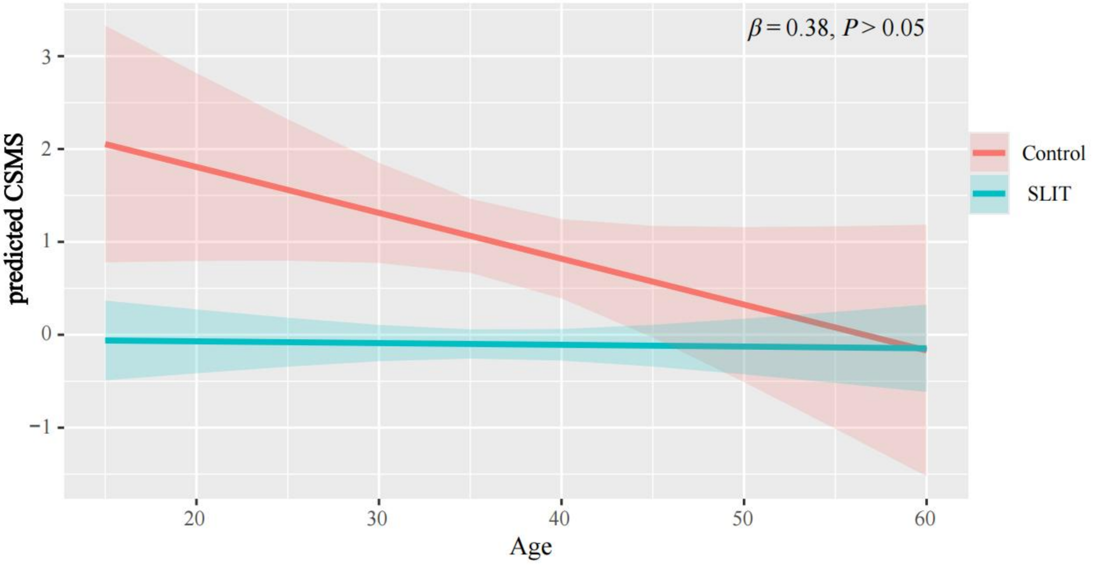

**Figure S2:** LMM-predicted TNSS over age for SLIT and control groups. LMM, linear mixed model; TNSS, total nasal symptom score; SLIT, sublingual immunotherapy.

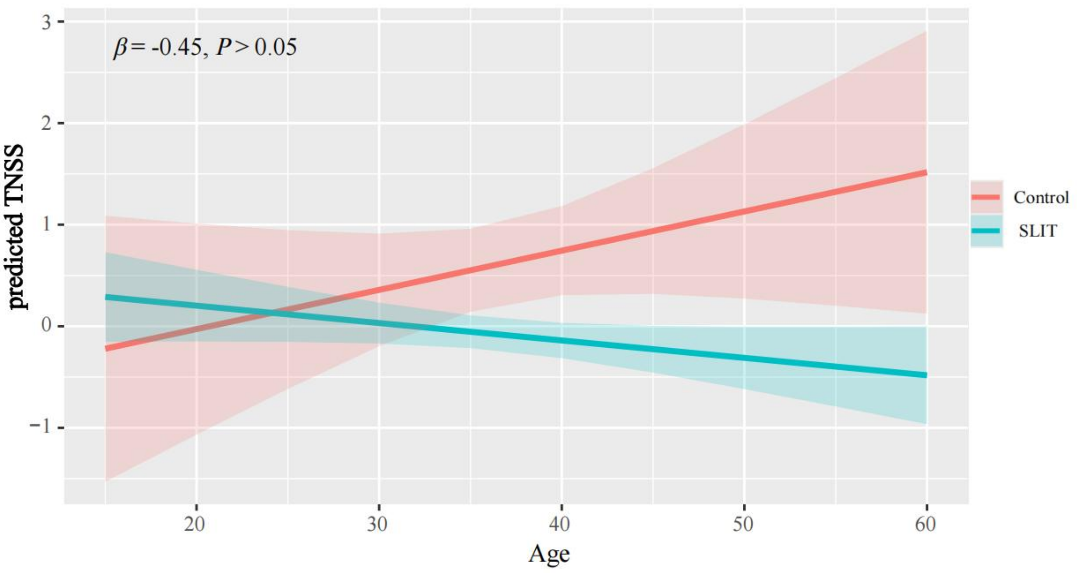

**Figure S3:** LMM-predicted TOSS over age for SLIT and control groups. LMM, linear mixed model; TOSS, total ocular symptom score; SLIT, sublingual immunotherapy.

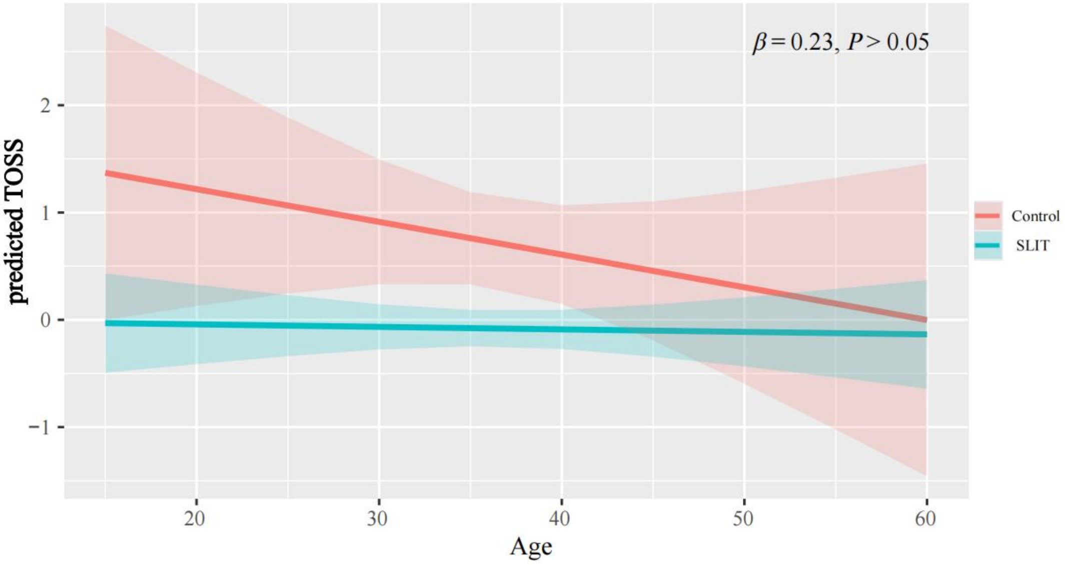

**Figure S4:** LMM-predicted MS over age for SLIT and control groups. LMM, linear mixed model; MS, medication score; SLIT, sublingual immunotherapy.

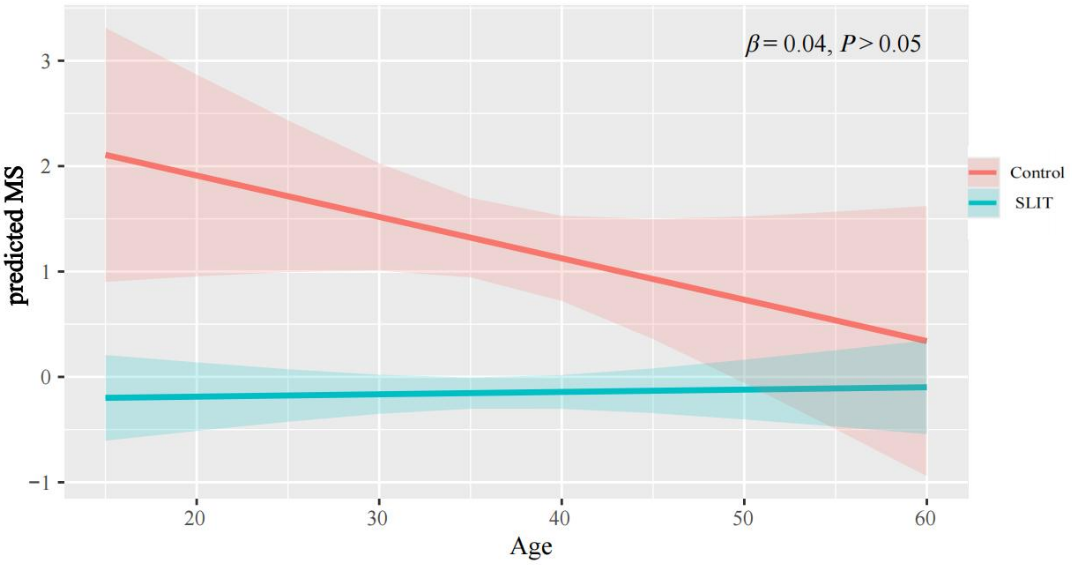

**Figure S5:** Efficacy comparison between male and female in SLIT group. CSMS, combined symptom-medication score; TNSS, total nasal symptom score; TOSS, total ocular symptom score; MS, medication score; SLIT, sublingual immunotherapy.

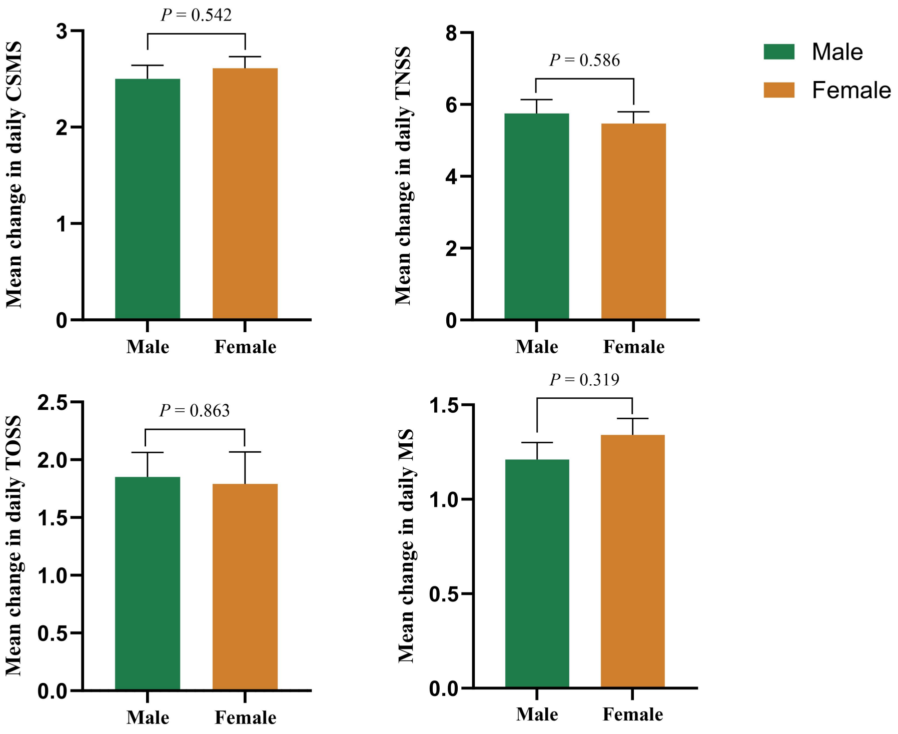


.
